# Impact of COVID-19 on TB and HIV/AIDS severity in low- and middle-income countries: a systematic review

**DOI:** 10.1016/j.dialog.2026.100349

**Published:** 2026-09-11

**Authors:** Endawoke Amsalu, Melese Yeshambaw, Dagim Jirata

**Affiliations:** a Armauer Hansen Research Institute, Ministry of Health, 1005 Jimma Road, Addis Ababa, Ethiopia; b Addis Ababa Universities, Department of Microbial, Cellular and Molecular Biology, Addis Ababa, Ethiopia

**Keywords:** COVID-19, LMICs, TB, HIV/AIDS, Severity, Pandemic, China

## Abstract

**Background:**

Beyond its profound health, social, and economic consequences, COVID-19 poses a serious threat to the management of tuberculosis (TB) and HIV/AIDS. However, empirical evidence remains limited. This study evaluates the impact of the COVID-19 pandemic on TB and HIV/AIDS service delivery across low- and middle-income countries.

**Methods:**

This systematic review protocol was registered with PROSPERO (CRD42021266242). Following PRISMA guidelines, we searched databases—including PubMed, ClinicalTrials.gov, and CINAHL—for studies on TB and HIV/AIDS services published between January 1, 2019, and January 8, 2024. A random-effects model calculated pooled relative risks to evaluate overall TB/HIV outcome severity, and subgroup analyses were performed to evaluate regional risk variations.

**Results:**

In low- and middle-income countries, non-TB patients with COVID-19 had a lower risk of severe outcomes compared to those with TB (pooled OR = 0.71, 95% CI: 0.60–0.83). Conversely, HIV patients with COVID-19 faced an increased risk of severe disease (pooled RR = 1.78, 95% CI: 1.45–2.19). By region, the pooled OR for severe COVID-19 in patients with TB was 0.83 (95% CI: 0.63–1.08) in Asia and 0.64 (95% CI: 0.51–0.79) in Africa. For HIV patients with COVID-19, the risk of severe disease was 1.73 (95% CI: 1.38–2.18) in Asia and 1.98 (95% CI: 1.26–3.10) in Africa.

**Conclusion:**

In low- and middle-income countries, COVID-19 patients with underlying conditions were significantly more likely to experience severe outcomes than those without comorbidities. Furthermore, the pandemic severely disrupted fragile health systems in these regions, exacerbating the burden of tuberculosis and HIV/AIDS.

## 1 Introduction

Coronavirus disease 2019 (COVID-19) has had enormous impacts on the economy, society, and healthcare since the onset of the virus in Wuhan, China [1]. During the early pandemic, the lockdown resulted in a significant impact on the economy and health system and influenced health services. For example, a significant impact of COVID-19 on maternal and neonatal outcomes was demonstrated, with increased adverse outcomes [2], [3]. Patients with underlying medical conditions are expected to experience adverse outcomes and complications from COVID-19 [4]. Moreover, several studies have associated the excellent effects of underlying conditions on SARS-CoV-2 outcomes [5]. Old age and comorbidities such as hypertension, diabetes, tuberculosis, HIV/AIDS, and cardiovascular disease are risk factors for the development of severe disease and a high mortality rate [6]. The underlying comorbidities that are significantly common in severe cases are cardiovascular disease, diabetes, chronic lung disease, malignancy, HIV and obesity [7], [8], [9], [10], [11], [12], [13], [14].

TB and HIV/AIDS remain leading causes of morbidity and mortality worldwide. Tuberculosis and HIV responses are threatened by COVID-19 [15], [16]. Concern remains that the pandemic could have a negative impact on the clinical course of TB and its ultimate outcome [17], [18], [19], [20], [21], [22], [23], [24]. However, only limited information about COVID-19 and active TB and HIV co-infection has been reported thus far, and those reported are in high-income regions [25]. Furthermore, several large cohort studies have reported the clinical characteristics and treatment outcomes of COVID-19 patients in the general population, and clinical outcome data concerning pandemics among patients with TB and HIV are sparse, especially in low- and middle-income countries. Currently, little evidence is available on the impact of COVID-19 on TB and HIV/AIDS services, detection, treatment and outcomes; however, the impact of COVID-19 on TB and HIV outcomes has not been well addressed for low- and middle-income countries with fragile health systems [18], [26]. Therefore, comprehensive evidence is needed on the effects of COVID-19 on TB and HIV services. The aim of this systematic review and meta-analysis was to determine the pooled risk effect of COVID-19 on TB and HIV/AIDS severity in low- and middle-income countries.

## 2 Methods

This study was registered with the International Prospective Register of Systematic Reviews (PROSPERO) with the registration number **CRD42021266242**. The Preferred Reporting Items for Systematic Reviews and Meta-Analyses (PRISMA) guidelines were used to review and report the results of this study [27].

### 2.1 Search strategy, selection criteria, and data extraction

The literature search was conducted from 2019 to 2024. Studies that were conducted in LMICs and published in any language from 2019 to 2024 were considered eligible for this review. Original studies providing information on the impact of COVID-19 on TB and HIV/AIDS severity identified through a computerized systematic search using PubMed, Google scholar, Medline, ClinicalTrials.gov, International Clinical forum, COVID-19 databases, and CINAHL. Terms within the same concepts were connected with the Boolean “OR” and combined with other search terms via the Boolean “AND”. The final search term was built in the PubMed database. The search included the following relevant medical subject heading terms: COVID-19, COVID-2019, 2019-nCoV, coronavirus, tuberculosis, TB, HIV, and outcome (Appendix 1). We also used a combination of keywords and search terms to identify studies from other databases, such as Google Scholar. References of identified studies were also searched to avoid the exclusion of relevant articles. After removal of duplicates via Endnote software, three levels of screening, namely, title, abstract and full-text review, were performed. After the removal of duplicate studies, those that did not fulfil the criteria were excluded. A detailed full-text review was conducted to identify potential articles reporting the effects of COVID-19 on TB and HIV outcomes. Studies identified with intended outcome interest judged to be of high quality were included in the analysis on the basis of the JBI quality appraisal checklist.

### 2.2 Exclusion criteria

Studies were excluded if the following conditions were not met: citation of abstracts, reports of unidentified sources, duplicate studies, case reports or studies that did not report outcomes among TB patients with COVID-19 to patients without COVID-19. Studies that include the impact of COVID-19 on TB and HIV/AIDS severity, anti-TB treatment and antiretroviral treatment for HIV patients have provided effective estimates of the relationship between COVID-19 and at least one of the severe TB outcomes (death, failure, relapse, drug resistance, and loss to follow-up). For HIV/AIDS, studies that involved HIV/AIDS patients and provided an effect estimate of the relationship between COVID-19 and at least one of the HIV/AIDS severity outcomes (death, lost to follow-up, opportunistic infections, stopped, viral load) and overall HIV/AIDS services, detection, and outcomes were included.

#### 2.2.1 Operational definition for outcomes

The main outcome of this study was disease severity. Severity of COVID-19 refers to cases when they are severe, critical, ICU, invasive mechanical ventilation, intubation or death. Secondary outcome other than severity such as services were also considered as the secondary outcome and examined in the narrative review.

### 2.3 Data extraction and review process

All of the research articles that were identified from searches of the electronic databases were imported into EndNote x8 software, and duplicates were removed. Before the data extraction, the full-length articles of the selected studies were read to confirm the fulfilment of the inclusion criteria. Data extraction was performed by two author's Melese Yeshambaw (and) Endawoke Amsalu independently. When there was a disagreement in data extraction between the reviewers, a third person Dagim Jirrta () was involved in the final decision. The selected studies were reviewed to extract data such as author name, year of publication, sample size, study design, study region/area, age, and variables that contain relevant information.

### 2.4 Methodological quality assessment

Two authors (Dagim Jirrta and Endawoke A) independently assessed the methodological quality of the included studies. The Joanna Bigger Institute (JBI) critical appraisal checklist for observational studies was used to assess the quality of the included studies. The JBI critical appraisal checklist for cohort and cross-sectional studies was used. After the quality of each included study was assessed on the basis of the critical appraisal checklist, the studies were judged to be of high quality and included in the meta-analysis (Table 1 and 2).

**Table 1:** Impact of COVID-19 on TB and HIV/AIDS severity outcome in low-and middle-income countries,2024.

					Non-TB		TB	
Author	Country	Study Design	Year	Grand	Event	Total	Event	Total
Bepouka BI, et al	Congo	Retrospective	2020	141	41	140	0	1
Wang D, et al.	China	Retrospective	2020	138	36	136	0	2
Wang W, et al.,	China	Retrospective	2020	421	59	417	0	4
Theresa, et al.,	Philippian	Longitudinal	2020	430	46	358	25	72
Chen, T., et al.,	China	Case series	2020	55	18	54	0	1
Du, R.H., et al.,	China	Prospective	2020	158	21	150	0	8
Gupta, N., et al.,	India	Retrospective	2020	21	5	18	3	3
Ibrahim,O.R.,et al.,	Nigeria	Retrospective	2020	144	6	143	1	1
Li, X., et al.,	China	Ambi-spective	2020	548	265	539	4	9
Lu Y, et al.,	China	Retrospective	2020	77	37	76	0	1
Maciel, et al.	Brazil	Cross-sectional	2020	416	217	415	0	1
Mo P, et al.,	China	Retrospective	2020	155	82	152	3	3
Nachega, J.B.,	Congo	Cohort study	2020	766	187	747	4	19
Yu Chen, et al.,	China	Case–control	2020	36	11	28	2	8
Zhang, J. J., et al.,	China	Retrospective	2020	140	56	138	2	2
Zhanng J, et al.,	China	Retrospective	2020	901	526	888	9	13
Biccard	Africa	Prospective	2021	3045	1432	2995	30	50
Li S et al.	China	Retrospective	2021	2924	249	2872	8	52
Zhang YT, et al.	China	Retrospective	2020	1350	226	1345	3	5
Xiao KH et al.	China	Retrospective	2020	143	35	139	1	4
Liu SJ et al.	China	Retrospective	2019	342	144	340	2	2
Liu J	China	Retrospective	2020	1033	152	1018	5	15

Severity (Severe, critical, Intensive care unit [ICU] admission, Invasive mechanical ventilation [IMV], intubation or death). TB: Tuberculosis. ** A multi-centres study in Africa that represents almost 10 countries. Event: Case(COVID-19).

**Table 2:** Impact of Covid-19 on HIV/AIDS severity in low-and middle-income countries,2024.

					HIV		Non-HIV	
Author	Year	Country	Design	Total	Event	Total	Event	Total
Alen G, et al.,	2021	Ethiopia	Retrospective	6	1	1	0	5
Biccard, et al.,	2020	Africa	Prospective	3084	149	1460	86	1587
Duncan, et al.,	2020	Zambia	Cohort study	443	61	321	17	122
Guan, et al.,	2020	China	Case control	1590	0	3	50	1587
Wu J, et al.,	2020	China	Retrospective	280	1	83	0	197
Huang, et al.,	2021	China	Retrospective	50,368	2	35	3869	50,333
Ombajo, et al.,	2020	Kenya	Cohort study	787	11	53	96	734
Nachega JBI, et al.,	2020	Congo	Cohort study	767	3	192	9	575
Shi, P et al.	2021	China	Retrospective	104	1	58	0	46

Severity (Severe, Critical, Intensive care unit [ICU] admission, Invasive mechanical ventilation [IMV], intubation or death). *Multi-county study of five Event: Cases(COVID-19).

### 2.5 Data analysis

Statistical analyses were carried out via STATA Version 14 software to compute the pooled risk for the effect of COVID-19 on TB and HIV severity. A narrative analysis was conducted to narrate the impact of the pandemic on TB/HIV services, case findings, detection, outcomes, and clinical presentations. Subgroup analysis based on study regions and services (TB and HIV in separate ways) was performed via forest plots with 95% CIs to estimate the pooled outcome of interest in LMICs. A meta-analysis using a random effects model was also used to assess the effects of the pandemic on TB/HIV severity. A random-effects estimate of the pooled OR (odd ratio) was generated for each outcome via the Mantel–Haenszel method. The chi-square *I*^*2*^ test was used to assess heterogeneity, with 25%, 50%, and 75% indicating low, moderate, and high heterogeneity, respectively. A sensitivity analysis was performed to test the robustness of the overall results when individual studies were excluded. Potential publication bias was assessed with Egger's test and funnel plots for visual inspection when sufficient studies (*n* > 10) were available.

## 3 Results

### 3.1 Characteristics of the included studies and participants

This review included prospective, retrospective, and case series studies. For TB synthesis, 23 studies were eligible, and 16 studies for which comparable outcomes were reported in at least one other study were included in the meta-analysis. Table 1 summarizes the characteristics of the included studies in LMICs, 2024. In this review, 13384 clients were included in the studies, with sample sizes ranging from 21 in India to 3045 in China. Similarly, for the review of HIV/AIDS, 57429 clients were included in the studies ranging from six in Ethiopia to 50,368 in China.

Fig. 1 shows the relative risk with 95% confidence intervals around the summary estimate. An increased risk of severe outcome was observed among non-TB patients with COVID-19, with a pooled OR of 0.71(95% CI: 0.60—0.83), compared with those with TB in low- and middle-income countries. Subgroup analysis by region revealed that the OR for severe TB in patients with COVID-19 were 0.83 (95% CI: 0.63, 1.08) and 0.64 (95% CI: 0.51, 0.79) for Asia and Africa, respectively. The proportion of severity of cases among Non-TB patients with COVID-19 was 21%. The proportions of severity of cases among TB patients with COVID-19 were 33%.

**Fig. 1:**
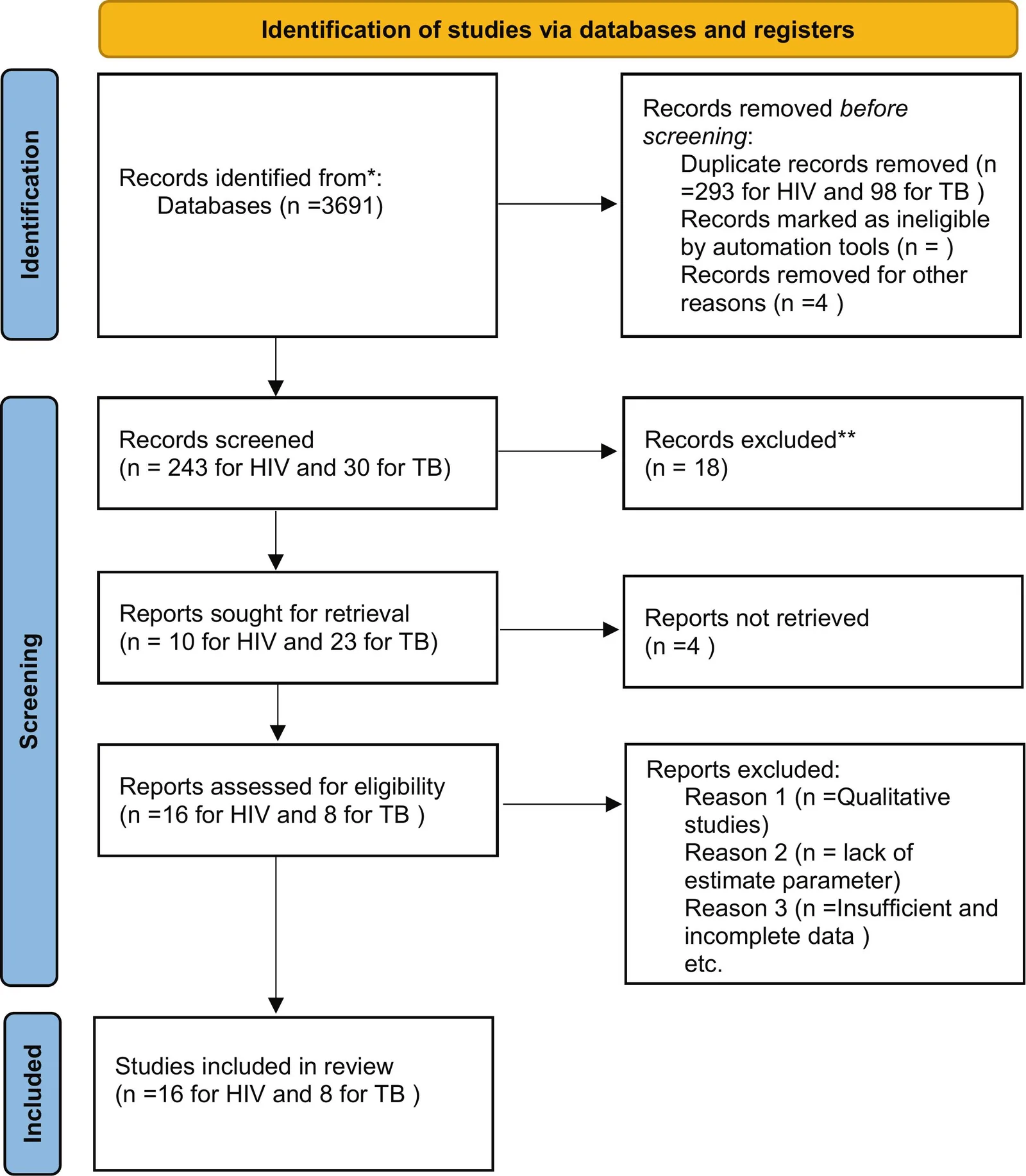
PRISMA flow chart for the impact of COVID-19 on TB and HIV/AIDS severity in LMICs, 2026.

### 3.2 Impact of COVID-19 on TB services (case findings, detection, clinical presentation & outcome)

The COVID-19 pandemic has overwhelmed tuberculosis health systems, severely compromising service access, active case finding, diagnostic detection, clinical management, and overall patient outcomes [28]. For example, the TB notification rate has decreased drastically by 60% in Ningxia, China [26]. In developing countries such as Africa, TB detection has decreased significantly [29], [30]. Poor adherence to TB and HIV medication severely impairs access to relief, reduces preventive therapy usage, hinders patient follow-up, and drives both drug resistance and the severity of mental health disorders. At the same time, TB service provision suffers from critical shortages in equipment, capacity, and non-medical patient support, such as counselling and food supplementation. These service interruptions are heavily exacerbated by COVID-19 mitigation measures, including movement restrictions, budget reallocations, and sudden funding cut-offs. [31]. TB control programs faced severe strain during the pandemic as resources, staff, laboratories, and Drug-Resistant TB (DR-TB) centres were diverted to COVID-19 relief. Combined with movement restrictions, these shifts compromised the diagnosis and treatment of both TB and TB/COVID-19 co-infections. Additionally, both conditions triggered widespread stigma, discrimination, and productivity losses. Decreased healthcare access, rising poverty, and post-COVID-19 lung complications further elevated global susceptibility to TB—factors that could accelerate disease progression and deteriorate treatment outcomes. While predictive models suggest these heightened risks are likely, concrete empirical evidence remains limited.

#### 3.2.1 Impact of COVID-19 on TB services (clinical presentation, detection, & outcome)

Following the COVID-19 outbreak, tuberculosis (TB) notification rates dropped sharply in early 2020 compared to the preceding 2–5 year averages. Declines were particularly steep in high-burden countries, including India (78%), China (47.8%), Uganda (43%), and Nigeria (34%). [25], [32], [33], [34], [35], [36]. Significant declines in TB screening, follow-up, and treatment uptake present severe challenges to disease control. Delayed diagnoses and undetected cases are projected to elevate transmission rates, with TB-related mortality expected to rise by 20% over the next five years. [37]. In a survey conducted in 106 countries, 78% of TB programs reported widespread disruption of TB services because of the pandemic [38]. Twenty high-burden TB countries further reported that TB program staff were engaged in pandemic-related activities, disrupting TB services [39]. New TB cases and mortality are affected by long-term increases due to the disruption of services. For example, a 3-month suspension of TB services could result in 19 million TB cases and 361,000 TB deaths in India and 24,700 TB cases and 12,500 deaths in Kenya [40]. Undetected tuberculosis (TB) cases and service disruptions—driven by limited support and medication stock-outs—jeopardized care during the pandemic. These bottlenecks heightened the risk of treatment delays and non-adherence, likely worsening therapeutic outcomes. Given the protracted nature of TB regimens, comprehensive outcome data remain delayed. Early evidence shows heterogeneous results: private-sector cohorts in Pakistan, China, Ethiopia, and Zimbabwe experienced a 5–15% relative decline in treatment success, whereas data from China, Kenya, and Malawi demonstrated no significant reduction. Separately, critically ill pandemic patients in African nations suffered higher mortality than those in Asia, Europe, and the Americas, driven primarily by severe critical care resource constraints. [41]. Patients co-infected with tuberculosis (TB) and COVID-19 exhibit clinical symptoms nearly identical to standalone cases, predominantly presenting with fever, breathlessness, and a persistent cough. Because both conditions carry significant social stigma, many individuals delay seeking medical attention, mistaking their TB symptoms for COVID-19 or avoiding diagnostic facilities out of fear. This reluctance created hidden transmission pipelines during strict lockdown periods; once movement restrictions eased, healthcare providers faced a sudden surge in diagnosed cases that severely overwhelmed an already strained medical infrastructure [33]. These selected studies were performed in regions with various demographic characteristics in developed and developing countries, mainly in China. The total number of participants in each study ranged from 24 to 203. The case fatality rate of patients with TB and Covid-19 co-infection was high (12.3%), while a combined diagnosis of TB and Covid-19 was found in 18.3% of patients [42]. This condition is linked to several complications, such as the need for an oxygen supply, pneumothorax, and extreme hypoxia. Research on BCG vaccination has shown that countries without vaccination policies are more likely to be affected seriously than BCG-vaccinated countries are. COVID-19 infection in patients with TB or a lack of sufficient BCG vaccination may be associated with greater detrimental consequences, including mortality [42].

### 3.3 Impact of COVID-19 on HIV services (detection, clinical presentation & outcome)

The COVID-19 pandemic severely disrupted HIV services, negatively impacting case detection, service uptake, and patient outcomes. Although comprehensive data on HIV services for patients co-infected with COVID-19 remain sparse, a World Health Organization (WHO) report noted that between April and June 2020, approximately 73 countries faced potential antiretroviral therapy (ART) disruptions, and putting 17.7 million people at risk. Limited access to care compromised adherence to TB, and HIV treatments, resulting in virological failure, drug resistance, and exacerbated mental health disorder. Consequently, uptake declined across key preventive measures, including post-exposure prophylaxis (PEP), BCG vaccination, and treatment-as-prevention programs [43]. Clinical profiles—including CD4 counts, viral loads, and time since HIV diagnosis—were similar between HIV patients with and without COVID-19, with no significant differences observed between those who recovered and those who died. Most patients were virally suppressed on antiretroviral therapy (ART). The most common presenting symptoms were cough, fever, and shortness of breath. [44]. HIV patients with COVID-19 tend to have moderate or severe disease outcomes. There is also concern that HIV-positive patients with COVID-19 are likely to have severe outcomes. Studies have also suggested that the examined risk factors for severe disease among HIV patients with COVID-19 infection are similar to those reported in COVID-19-non-HIV groups [45], [46].

Fig. 2 shows the effect estimate expressed as an odds ratio for the impact of COVID-19 on HIV/AIDS outcomes in low- and middle-income countries in 2026. The effect estimate expressed as a percentage in the figure below shows that COVID-19 increased HIV/AIDS outcomes. In the same graph, the effect estimate expressed as a proportion shows the increased outcomes of HIV/AIDS due to COVID-19 across contents. The ORs of increased severity of HIV/AIDS due to COVID-19 were 1.73 (95% CI: 1.38; 2.18) and 1.98 (95% CI: 1.26; 3.10) in African and Asian countries, respectively. The proportion of severity of COVID-19 on HIV was 90% and non-HIV was 3%, respectively.

**Fig. 2:**
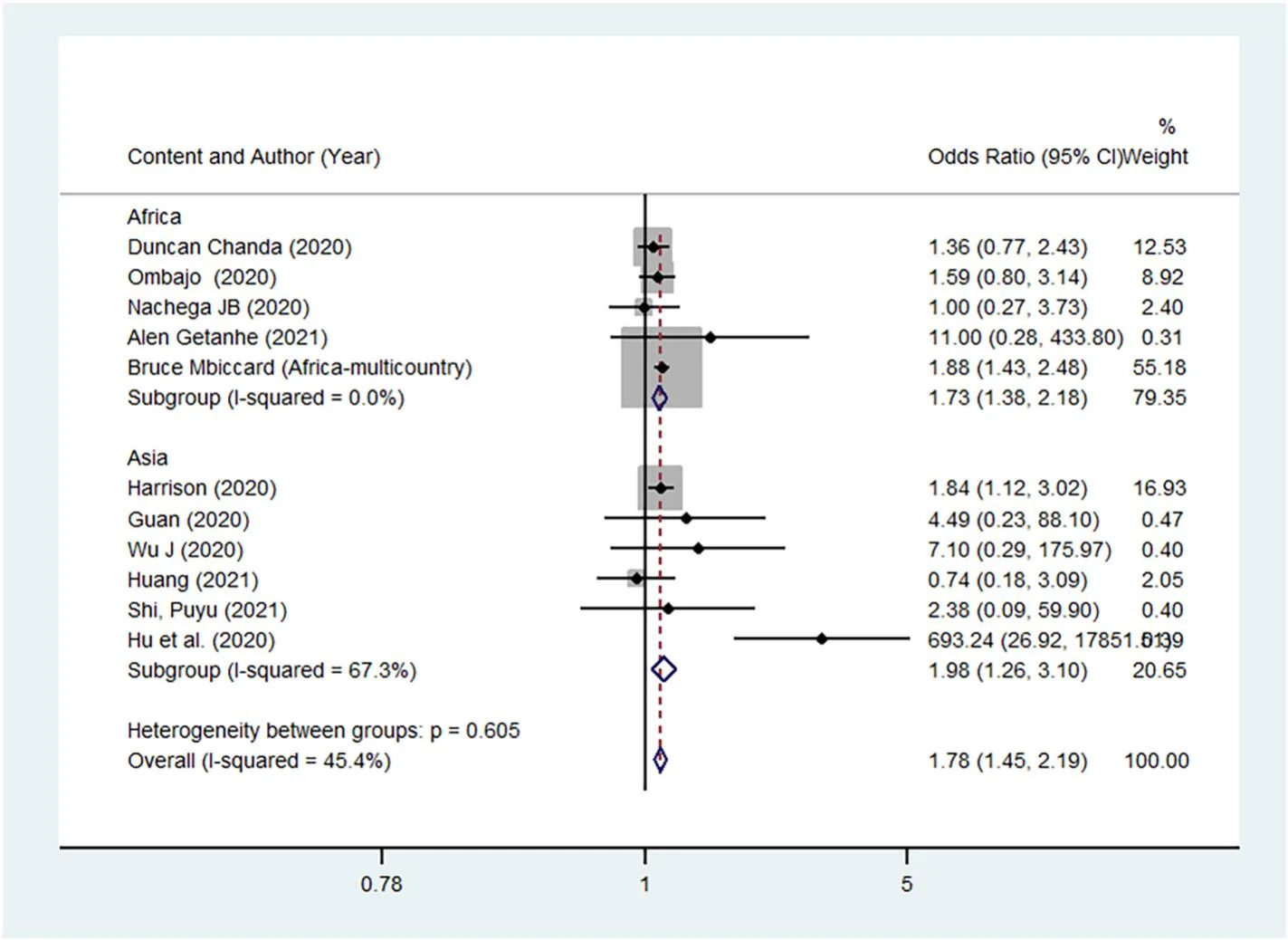
Forest plot for the impact of COVID-19 on HIV/AIDS outcomes (Odd ratio) in low- and middle-income countries, 2026.

### 3.4 Publication bias

Fig. 3 below show the publication bias and funnel plot. The finding of sensitivity analysis shows the effect estimate was robust. There is no publication bias. The egger test value indicated show that the 0.0044 with insignificant *p*-value (Appendix).

## 4 Discussion

Our comprehensive review of 17,177 TB and 110,167 HIV clients in low- and middle-income countries highlights major pandemic disruptions to service delivery, case finding, detection, and patient outcomes. COVID-19 infection rates reached 10% among people living with HIV and 33% among TB patients. While clinical presentations (primarily fever, cough, shortness of breath, and fatigue) remained similar regardless of COVID-19 status, co-infection significantly increased the risk of severe TB and HIV outcomes [26]. Multisite prospective cohort studies in African countries have shown that the clinical picture of COVID-19 patients with TB and without TB has not shown a visible clinical picture.

Here, we show that COVID-19 patients with TB and HIV have an increased risk of severe disease. Recently, a rapid systematic review in China by Gao et al. demonstrated an increased risk of severe patients with underlying conditions compared with those who were not [18]. Conversely, other studies show that TB/HIV co-infected patients share a similar risk of contracting COVID-19 as non-co-infected individuals. A recent narrative review similarly found no significant difference in severe disease risk between COVID-19 patients with and without HIV. Within this cohort, TB patients with COVID-19 had a 29% lower pooled risk of severe outcomes compared to those without COVID-19 (RR = 0.71), though nearly one-third of the TB/COVID-19 group still experienced severe outcomes. However, a recent systematic review highlighted that COVID-19 significantly elevated overall TB mortality (RR = 2.10; 95% CI: 1.75–2.51). [47]. Similarly, a recent systematic review and meta-analysis reported that TB was associated with an increased risk of severe COVID-19 [48]. Another study by Chen et al. revealed an increased risk of severe cases among the tuberculosis with COVID-19 group [5]. However, Du et al. reported that non- survivors had a lower prevalence of TB than survivors did [17]. A multi-center cohort study reported a high case fatality rate (12.3%) among patients with tuberculosis and COVID-19 [49]. Furthermore, a study conducted by Motta et al. revealed that the case fatality rate among tuberculosis patients with COVID-19 was approximately 11.6% [50].

This review demonstrated that the prevalence of COVID-19 among TB patients varies by country. Similarly, a study illustrated that the variation in the infection rate in TB groups varies by WHO study region in high-, low-, and very low-burden countries [51]. Despite preventive measures, innovations for effective treatment, and vaccine coverage, many studies have shown that TB health services have decreased patient follow-up, testing and prevention coverage, and treatment outcomes. Therefore, there is a need to ensure adequate treatment for known TB via the provision of additional medicine and digital treatment support. The first requires a good understanding of the effect of COVID-19 on health services and the resources available and required to restore these resources to at least pre-pandemic levels. In addition, there is a need to understand how vulnerability to TB and *M. tuberculosis* transmission has changed. Studies have also provided strong support for data on delays in diagnosis and treatment, changes in patients' costs for TB services, the impact of COVID-19 infection and disease vulnerability on TB and mortality, and the effects of changes in contacts and mobility on household and community transmission of TB. COVID-19 disaggregated TB health service availability and the need for frequent reporting.

This study revealed the impact of COVID-19 among HIV/AIDS groups with respect to ART treatment, prevalence, services and outcomes. Accordingly, the rate of new HIV infection among COVID-19 patients ranges from 0.8% to 1.8% according to PCR tests in high-income countries [52]. In China, the rate of new HIV infection via PCR and clinical diagnosis was approximately 0.68% in COVID-19 patients. The co-infection rate in this review slightly varied from that reported in other studies [44], [53], [54], [55], [56], [57]. We found that the prevalence of new HIV infections was approximately 10% among COVID-19 patients. The rate of infection among TB and HIV patients should be considered with caution, as there may be repeated infections among those with known underlying conditions, and as there may be a fear of acquiring COVID-19 infections, patients may undergo repeated tests. This review revealed that the risk of developing severe disease among HIV-positive patients with COVID-19 was 1.78 times greater than that among non-HIV-infected patients. However, a recent review conducted by Sarkar and Hariyan et al. on the impact of COVID-19 on concurrent infections revealed that COVID-19 has no significant effect on HIV outcome [47], [57]. A review conducted by Hariyaa et al. revealed that a subgroup analysis involving only studies from Africa revealed that HIV was associated with poor composite outcomes [OR 1.11 (95% CI 1.03–1.21)] [57]. Although most studies reported that HIV patients with COVID-19 were on ART and had well-controlled HIV infection, some studies reported poor outcomes among immune-deficient patients and immunosuppressed patients with COVID-19 [58]. Similarly, a study conducted by Grvasoni et al. reported that 28% of people living with HIV required hospitalization, with a single incidence of death [59].

Another population-based cohort study from the UK reported that PLHIV had a greater risk of COVID-19 death than did those without HIV (HR = 2.90; 95% CI, 1.96–4.30)0.55 PLHIV should not be considered protected from SARS-CoV-2, as people with low CD4+ T-cell counts may have poorer outcomes than people with normal immunity [47]. Moreover, most PLWH co-infected with COVID-19 have mild to moderate disease [60], [61], [62] [41, 43, 44, 49, 51–56]. However, two studies reported that the majority of COVID-19 cases among PLWH were severe and associated with a high mortality rate [63].

The strengths of this review include that the comprehensive search was not restricted by language and that a broad range of literature was included and synthesized. Additionally, this was the first study to analyse the impact of COVID-19 on tuberculosis and HIV/AIDS in low- and middle-income countries. The main limitations of this study were the retrospective design of the included studies, as well as the heterogeneity of the study populations and the definitions and ways of measuring outcomes, thereby limiting the comparability of the results. Finally, we could not exclude the risk of publication bias against studies reporting negative findings. Because of the lack of data, this study did not comprehensively compare the risk estimates between the two groups in terms of risk factors and the clinical presentation of TB patients with COVID-19 because of fragmented data. Therefore, further studies should encompass the risk factors for severe outcomes, as more studies are expected to be generated.

## Availability of data and materials

All data generated or analysed during this study are included in this published article.

## CRediT authorship contribution statement

**Endawoke Amsalu:** Formal analysis, Data curation, Conceptualization. **Melese Yeshambaw:** Writing – review & editing, Methodology, Data curation. **Dagim Jirata:** Writing – review & editing, Writing – original draft, Visualization, Validation.

## Funding

The authors declare that they have no received any financial funding.

## Declaration of competing interest

The authors declare that they have no competing interests.
