# Supplementary material for: Impact of COVID-19 on TB and HIV/AIDS severity in low- and middle-income countries: a systematic review
Source: Dialogues Health. 2026 Sep 11;9:100349. doi: 10.1016/j.dialog.2026.100349 (PMC13632020; doi:10.1016/j.dialog.2026.100349)
Supplement: Supplementary file 1 [file mmc1.docx]

**Appendix**

**PubMed only**

I. **For Tuberculosis:**

*("COVID-19"[All Fields] OR "COVID-2019"[All Fields] OR "severe acute respiratory syndrome coronavirus 2"[Supplementary Concept] OR "severe acute respiratory syndrome coronavirus 2"[All Fields] OR "2019-nCoV"[All Fields] OR "SARS-CoV-2"[All Fields] OR "2019nCoV"[All Fields] OR (("Wuhan"[All Fields] AND ("coronavirus"[MeSH Terms] OR "coronavirus"[All Fields])) AND (2019/12[PDAT] OR 2020[PDAT]))) AND ("tuberculosis"[MeSH Terms] OR "TB"[All Fields]), which was the primary search engine used.*

II. **For HIV/AIDS:**

*("COVID-19"[All Fields] OR "COVID-2019"[All Fields] OR "Severe acute respiratory syndrome coronavirus 2"[Supplementary Concept] OR "Severe acute respiratory syndrome coronavirus 2"[All Fields] OR "2019-nCoV"[All Fields] OR "SARS-CoV-2"[All Fields] OR "2019nCoV"[All Fields] OR (("Wuhan"[All Fields] AND ("coronavirus"[MeSH Terms] OR "coronavirus"[All Fields])) AND (2019/12[PDAT] OR 2020[PDAT]))) AND ("HIV"[MeSH Terms] OR "Human immune deficiency virus"[All Fields]). A combination of keywords and search terms was used to identify studies from the Google Scholar database. References of identified studies were searched to avoid the exclusion of relevant articles. All identified articles from the different databases were imported into Endnote reference management software.*

Fig 1: funnel plot for the impact of COVID-19 on Tuberculosis severity in low-and middle income countries, 2024.
