# Supplementary material for: Impact of COVID-19 on TB and HIV/AIDS severity in low- and middle-income countries: a systematic review
Source: Dialogues Health. 2026 Sep 11;9:100349. doi: 10.1016/j.dialog.2026.100349 (PMC13632020; doi:10.1016/j.dialog.2026.100349)
Supplement: Supplementary file 2 [file mmc2.docx]

**Identification of studies via databases and registers**

Records removed *before screening*:

Duplicate records removed (n =293 for HIV and 98 for TB )

Records marked as ineligible by automation tools (n = )

Records removed for other reasons (n =4 )

Records identified from*:

Databases (n =3691)

**Identification**

Records screened

(n = 243 for HIV and 30 for TB)

Records excluded**

(n = 18)

Reports sought for retrieval

(n = 10 for HIV and 23 for TB)

Reports not retrieved

(n =4 )

**Screening**

Reports assessed for eligibility

(n =16 for HIV and 8 for TB )

Reports excluded:

Reason 1 (n =Qualitative studies)

Reason 2 (n = lack of estimate parameter)

Reason 3 (n =Insufficient and incomplete data )

etc.

Studies included in review

(n =16 for HIV and 8 for TB )

**Included**

Figure 1: PRISMA flow chart for the impact of COVID-19 on TB and HIV/AIDS severity in LMICs, 2026.

Source: Page MJ, et al. BMJ 2021;372:n71. doi: 10.1136/bmj.n71.

This work is licensed under CC BY 4.0. To view a copy of this license, visit <https://creativecommons.org/licenses/by/4.0/>
